# Health 2.0 and Medicine 2.0: Tensions and Controversies in the Field

**DOI:** 10.2196/jmir.1056

**Published:** 2008-08-06

**Authors:** Benjamin Hughes, Indra Joshi, Jonathan Wareham

**Affiliations:** ^2^West Hertfordshire Hospitals NHS Trust (WHHT)Hemel HempsteadUK; ^1^Department of Information SystemsESADEBarcelonaSpain

**Keywords:** Web 2.0, Medicine 2.0, Health 2.0

## Abstract

**Background:**

The term *Web 2.0* became popular following the O’Reilly Media Web 2.0 conference in 2004; however, there are difficulties in its application to health and medicine. Principally, the definition published by O’Reilly is criticized for being too amorphous, where other authors claim that Web 2.0 does not really exist. Despite this skepticism, the online community using Web 2.0 tools for health continues to grow, and the term *Medicine 2.0* has entered popular nomenclature.

**Objective:**

This paper aims to establish a clear definition for Medicine 2.0 and delineate literature that is specific to the field. In addition, we propose a framework for categorizing the existing Medicine 2.0 literature and identify key research themes, underdeveloped research areas, as well as the underlying tensions or controversies in Medicine 2.0’s diverse interest groups.

**Methods:**

In the first phase, we employ a thematic analysis of online definitions, that is, the most important linked papers, websites, or blogs in the Medicine 2.0 community itself. In a second phase, this definition is then applied across a series of academic papers to review Medicine 2.0’s core literature base, delineating it from a wider concept of eHealth.

**Results:**

The terms *Medicine 2.0* and *Health 2.0* were found to be very similar and subsume five major salient themes: (1) the participants involved (doctors, patients, etc); (2) its impact on both traditional and collaborative practices in medicine; (3) its ability to provide personalized health care; (4) its ability to promote ongoing medical education; and (5) its associated method- and tool-related issues, such as potential inaccuracy in enduser-generated content. In comparing definitions of Medicine 2.0 to eHealth, key distinctions are made by the collaborative nature of Medicine 2.0 and its emphasis on personalized health care. However, other elements such as health or medical education remain common for both categories. In addition, this emphasis on personalized health care is not a salient theme within the academic literature. Of 2405 papers originally identified as potentially relevant, we found 56 articles that were exclusively focused on Medicine 2.0 as opposed to wider eHealth discussions. Four major tensions or debates between stakeholders were found in this literature, including (1) the lack of clear Medicine 2.0 definitions, (2) tension due to the loss of control over information as perceived by doctors, (3) the safety issues of inaccurate information, and (4) ownership and privacy issues with the growing body of information created by Medicine 2.0.

**Conclusion:**

This paper is distinguished from previous reviews in that earlier studies mainly introduced specific Medicine 2.0 tools. In addressing the field’s definition via empirical online data, it establishes a literature base and delineates key topics for future research into Medicine 2.0, distinct to that of eHealth.

## Introduction

O’Reilly defines Web 2.0 by a series of case examples, noting the characteristics of a Web 2.0 company, such as (1) hard-to-recreate data sources that get richer as more people use them, (2) harnessing collective intelligence, and (3) levering the “long tail” through customer self service [1]. However, critics have claimed this definition is too amorphous [2] and have attempted to narrow it [3]. Despite these attempts, researchers can view Web 2.0 in its widest sense, incorporating all tools such as search (eg, Google) and Podcasts [4,5]. Since many top websites [6] encompass some of these characteristics, such as use of RSS feeds, it poses a concern that Web 2.0 and the Internet are synonymous. Furthermore, existing research fields in medicine, such as interactive health communication applications (IHCAs), overlap significantly with components of Medicine 2.0. These ambiguities imply that Medicine 2.0 is not a separate research field. 

However, we argue that Medicine 2.0 has certain characteristics that warrant analysis distinct from eHealth. First, there is the number of online references to Web 2.0, Health 2.0, and Medicine 2.0 (187-224 million, 0.5-1.7 million, and 0.1-0.4 million, respectively, depending on the search engine used). Second, there is extensive literature loosely associated with O’Reilly’s definition, such as Wikinomics [7], Democratizing Innovation [8], or the literature identified in this review. Third, related topics such as IHCAs and eHealth either do not cover all aspects of Medicine 2.0 or have a different focus. For instance, IHCAs were defined before recent Internet developments such as Wikipedia, which is reflected in doubts about which sites apply to IHCAs [9]. Hence, we believe the main issue is that a clearer definition or demarcation of Medicine 2.0 is warranted.

We employ data garnered from practising online communities to answer the following research questions:

1. Can a clear definition of Medicine 2.0 be established across practitioner and academic literature that distinguishes this field from eHealth?
2. Is there agreement between online discussions and academic communities in their use of the term *Medicine 2.0*? If not, what does such divergence imply for future research?
3. What are the major tensions between the main stakeholders in Medicine 2.0 communities as identified by research?

Toward this aim, we used Google’s PageRank system to identify the most popular online discussions and delineate key themes through thematic analysis. We started by clarifying the Web 2.0 definition as some researchers suggest that aspects of its application to medicine cannot be assumed [10]. We then examined both academic literature and online discussions to find key identifying terms and salient themes associated with Medicine 2.0 (or other health “2.0”–related terms). Indeed, the Medicine 2.0 definition was found to be different from simply applying the rule “Medicine 2.0 = Medicine + Web 2.0,” particularly in its emphasis on personalized health care and its participants. In addition, we found only minor differences between the salient themes in Health 2.0 and Medicine 2.0.

In a second phase, we applied these salient themes as a definition to the academic literature associated with Medicine 2.0 to broadly delineate the field. In doing this, we found four major tensions in the field. Moreover, we determined that academic literature does not explore personalized or customized health care in the detail that this theme is treated online. Finally, as could be expected, we found a gray area with papers that clearly have implications for Medicine 2.0 but do not correspond to many of the salient themes associated with it.

This paper makes a distinct contribution to the Medicine 2.0 field by empirically demarcating its thematic boundaries and differentiating it from Web 2.0 and Health 2.0, as well as online versus academic perspectives.

## Methods

### Identifying Medicine 2.0 Salient Themes and Vocabulary

Medicine 2.0 focuses strongly on the use of Web 2.0 tools. However, as a term only four years old and constantly evolving as new tools emerge, academic literature is unlikely to have achieved consensus on its scope as quickly as 2008. For this reason, we used Google’s PageRank system to identify the tools or benefits most important to Web 2.0. Google’s PageRank relies on the democratic nature of the Web’s vast link structure to indicate an individual page’s value. Google interprets a link from page A to B as a vote by page A for page B. Google looks at more than the sheer volume of votes; if the page that casts the vote also has many links to it, this vote cast by that page weighs more heavily [11].

To refine the approach, and to enable a contrast to Medicine 2.0’s salient themes, we started with Web 2.0. We searched with Google for “Web 2.0” to identify the most linked pages with the term. These pages were coded using thematic analysis [12] to identify the terms describing both tool types and the purpose or benefits of Web 2.0. Articles coded included not only the Wikipedia entry and O’Reilly’s definitions, but also a series of blog threads, including over 50 contributions from users attempting to define Web 2.0. The full results of this approach are available in a separate publication [13], but we adapted the approach for this paper and used the result, which summarizes Web 2.0 as:

Democratized Collaborations; a collaboration enabled by web technology that promotes learning and innovation. Democratized collaborations work by connecting participants to harness network effects and knowledge in an open and interactive manner.

### Defining Medicine 2.0

A similar approach was used to delineate Medicine 2.0, but no assumption was made that identifying terms such as *Medicine 2.0* should take precedence over *Physician 2.0* as the researchers had a priori knowledge that numerous terms are associated with the field. Hence, in the first two steps in the method below, we try to determine the identifying terms that describe the field. Step 3 identifies the most popular online discussions relevant to the field, and, finally, steps 4-6 use the comparative method for thematic analysis as described by Techniques and Procedures for Developing Grounded Theory [12] to understand the salient themes. Steps 3-6 were effectively completed three times in order to obtain intercoder reliability of 82% agreement for exact phrases across all of the pages analyzed.

In a second phase, carried out in step 7, the original sample of 2405 academic papers identified as being potentially related to Web 2.0 and health was reduced to 56 papers after excluding those not directly addressing Medicine 2.0, duplicate search results, or papers not available in English. The initial number of papers and those selected for the review are shown in brackets in step 1 of the methodology outlined in Table 1. 

**Table 1:** Methodolocial steps

Step	Purpose	Description
1	Determine the field’s identifying terms from academic literature	We examined journals through search tools including PubMed (170:16), Blackwell Synergy (159:3), Science Direct (52:2), Emerald Insight (21:1), SpringerLink (20:1), JAMA (10:1), Wiley Interscience (109:0), and Google Scholar (1864:32). Any paper with a combination of “web” and “2.0” and restricted to medicine or health science journals was considered. The Google Scholar search was based on “Web 2.0” and “medicine” or “health.” All key “2.0” terms found in these paper titles or abstracts were identified (eg, “Medicine 2.0”). This and subsequent use of literature covers papers up to the end of March 2008.
2	Determine the popularity of academic literature’s identifying terms online	These terms were used to search Google to determine the support for the particular term (eg, the number of references matching “Health librarian 2.0”) online.
3	Determine the most popular pages associated with the identifying terms	Identifying terms with the most online references (eg, “Health 2.0” and “Medicine 2.0”) were used as a search term in Google to identify the most popular associated pages. Google’s PageRank system returns the most popular and most viewed pages as denoted by the richer-get-richer phenomena noted by a number of authors [14,15]. While these pages are the most popular, their contribution to the field may not be the most important [16], necessitating step 5.
4	Identify salient themes using thematic analysis	The online discussions in the popular pages were analyzed by two researchers using thematic analysis [12] to identify salient themes. This process involves open coding, axial coding, and selective coding in an iterative process of analyzing qualitative data (ie, text). Units of text (ie, words, phrases, sentences, or paragraphs) are labeled, compared, and grouped until no new categories emerge. Coders were instructed to look for manifest-type content that describes the field. Manifest content is that which resides on the surface of communication and is therefore easily observable, as this can improve reliability and puts less interpretative burden on coders [17]. As such, exact phrases that were found in the pages were used, though the unit of analysis combined both the exact phrase and the theme (an approach noted in studies such as [18]).
5	Identify order of importance of pieces of exact phrases associated with salient themes	As noted in step 3, the most popular pages do not necessarily make the only important contributions to define the field, even though they do potentially play a more important role than other pages. The exact phrases associated with the different salient themes identified were re-entered into four different search engines to understand their frequency of use online or their relative ranking.By ranking, we mean the frequency of use as indicated by the count function of the search engine compared to other phrases using the same search engine. The search text included the identifying term as set out in Table 2.In this way, we were able to identify the importance of this exact phrase across all online content, reducing reliance on the popular pages analyzed. Exact phrase within themes were excluded if they did not have minimum counts that met search engine reliability thresholds (eg, less than 1000 for Google, 8000 for Microsoft Live Search) [19,20].
6	Identify further salient themesuntil saturation	Additional online descriptions continued to be coded until saturation (eg, nine online articles were examined for Health 2.0, and the next two examined did not identify any phrases with over a 1000 counts online). At this point, the independent coders compared and returned to step 3, where required, to address interrater reliability and integrity.
7	Define field scope and review academic literature to determine related publications and key tensions	This understanding of salient themes and the frequency of use of exact pieces of text online was used to provide an updated definition of Medicine 2.0 and structure the academic literature into key themes. The original set of academic papers identified in step 1 was critically examined to determine if the papers were, in fact, Medicine 2.0, to clearly delineate between Medicine 2.0 and eHealth literature. Two researchers independently assessed the literature to determine if it was specific to Medicine 2.0. The differences were resolved by discussion between the two researchers. Key tensions were identified via discussions with the whole research team.

## Results

### Determine Field’s Identifying Terms

The abstracts and titles of the 2405 papers indicated that “2.0” was associated with Health 2.0, Medicine 2.0, Physician 2.0, Nursing Education 2.0, Medical Librarian 2.0, and Physician Learning 2.0.

### Determine the Popularity of Identifying Terms Online

Table 2 shows how often the terms used by academics are replicated in the community itself (via Google search). The results show that “Health 2.0” or “Health” and “Web 2.0” are the most commonly discussed terms. The prominence of Health 2.0 and Medicine 2.0 meant only these terms were examined for more precise definitions as detailed by steps 3-6 in the Methods.

**Table 2:** Online use of “2.0” terms identified in academic literature

Search Term	Google Count
“health” and ”web 2.0” or “health 2.0”	1,617,000
“medicine” and “web 2.0” or “medicine 2.0”	474,900
“physician 2.0” or “physician” and “web 2.0”	126,000
“medical librarian 2.0” or “medical librarian” and “web 2.0”	9560
“nursing education 2.0” or “nursing education” and “web 2.0”	5612
“physician learning 2.0” or “physician learning” and “web 2.0”	271

### Identify Salient Themes and Popularity of Associated Phrases

For both terms, open coding of the top online descriptions quickly lead to saturation, in the case of Medicine 2.0, after seven articles (articles coded: [4,21-25]) and after nine for Health 2.0 (articles coded: [26-34]). In the early axial and selective coding stages, four core terms were identified: participants or actors, tools, methods, and purpose or objectives. The salient themes or grouping applied to both identifying terms, and there was almost no difference with the ranking (in terms of counts) of exact phrases associated with these themes. Overall, there were few differences between Health 2.0 and Medicine 2.0 in terms of participants, and Table 3 and Table 4 show the individual counts for each term. It is worth noting than one exact phrase, “Privacy,” was identified by both researchers but was not possible to rank using search engines. Different search engines provided widely different rankings for this term (from first to last within the methods and tools grouping), which we believe reflected the fact that some search engines perform key word searchers through the footers of cached pages (see Discussion).

**Table 3:** Medicine 2.0: relative frequency of use of associated text

Salient Theme	Associated Exact Phrase	Ranking (relative frequency of use online)				
		Google	Yahoo!	MSN	Ask.com	Average Rank
Participants	Doctors, physicians	1	1	1	1	1
	Patients	2	2	2	2	2
	Scientists	3	3	3	3	3
	Nurses	4	4	4	4	4
	Medical students	5	5	5	5	5
	Medical librarians	6	6	6	6	6
Tools	Podcast	1	1	1	1	1
	Blog	2	2	2	2	2
	Bookmarking, tagging	3	3	5	4	3.75
	Search engine	4	4	6	5	4.75
	Wiki	5	5	3	6	4.75
	RSS feed	6	6	4	3	4.75
Methods	Commons, open access	1	1	1	1	1
	Wisdom of crowds, network effects	2	2	3	4	2.75
	User generated content	3	3	4	3	3.25
	Accuracy	4	4	2	2	3
	Expert community	5	5	5	5	5
Purpose/ Objectives	Collaborate, facilitate collaboration	1	1	1	1	1
	Personalized, customized information	2	2	2	2	2
	Medical education	3	3	3	3	3
	Free access, free services	5	4	4	4	4.25
	Stay informed	6	5	5	5	5.25
	Communication tool	4	6	6	6	5.5
	Create knowledge	7	7	7	7	7

**Table 4:** Health 2.0: relative frequency of use of associated text

Salient Theme	Associated Exact Phrase	Ranking (relative frequency of use online)				
		Google	Yahoo!	MSN	Ask	Average Rank
Participants	Doctors, physicians	1	1	1	1	1
	Patients, citizens	2	2	2	2	2
	Scientists	3	3	3	3	3
	Medical students	4	5	6	4	4.75
	Nurses	5	4	4	5	4.5
	Clinicians	6	6	5	6	5.75
	Health professionals	7	7	7	7	7
	Caregivers	8	8	8	8	8
	Medical librarians, health librarians	9	9	9	9	9
Tools	Blog	1	1	1	1	1
	Podcast	2	2	2	2	2
	Tagging, bookmarking, social search	3	3	6	3	3.75
	Search engine	4	4	4	4	4
	RSS feed	6	6	3	5	5
	Wiki	5	5	5	6	5.25
	Mashup	7	7	7	7	7
Methods	Open source, open platforms	1	1	1	1	1
	User generated, user innovation	2	2	2	2	2
	Participation, power of networks	3	3	3	3	3
	Aggregation	4	4	4	4	4
	Taxonomy	5	6	6	5	5.5
	Reliable information, medical errors	6	5	5	6	5.5
	Virtual communities, social groups	7	7	7	7	7
Purpose/Objectives	Long tail, personalized	1	1	1	1	1
	Collaboration	2	2	3	2	2.25
	e-learning, medical education, mobile learning, health education, active learning	3	3	2	3	2.75
	Community	4	4	5	4	4.25
	Online services	5	5	4	5	4.75
	Knowledge sharing	6	6	6	6	6
	Information infrastructure	7	7	8	7	7.25
	Reference tool	8	8	7	8	7.75

### Define Field Scope and Review Academic Literature

Given the similar definitions of Health 2.0 and Medicine 2.0, and as suggested by other authors to encapsulate research [35,36], we decided to use the term *Medicine 2.0.* However, choosing either term would not have highly impacted the results of the literature review. The ranking of the terms and the context of use in the pages that we analyzed suggested the following definition for Medicine 2.0:

Medicine2.0 is the use of a specific set of Web tools (blogs, Podcasts, tagging, search, wikis, etc) by actors in health care including doctors, patients, and scientists, using principles of open source and generation of content by users, and the power of networks in order to personalize health care, collaborate, and promote health education.

Supporting this are five salient or structuring themes that we more accurately define as follows:

1. Participants: the different stakeholders in Medicine 2.0
2. Method/tools: the manner by which Medicine 2.0 information is created and owned (eg, its accuracy from user generation, open source or ownership, and the use of specific tools such as wikis)
3. Collaboration and practice: Medicine 2.0 as a tool to promote participant’s interests as a reader (staying informed) or to communicate and collaborate collectively for his or her own practice
4. Medical education: Medicine 2.0’s educational use for the general public, training new health professionals, or ongoing education for specialists (different than collaboration and practice in its promotion of general skills, as opposed to examining and collaboration on a patient’s particular case)
5. Personalized health: Medicine 2.0 as a mechanism to provide customized health care, such as connecting patients with rare conditions, and to improve an individual’s value from health care

## Discussion

### Research Question 1: Definition

Can a clear definition of Medicine 2.0 be established across practitioner and academic literature that distinguishes this field from eHealth? Examining this question, we found common salient themes for both Health 2.0 and Medicine 2.0 that describe Web 2.0’s application to health. Its application to health and medicine is not as straightforward as the rule “Medicine 2.0 = Medicine + Web 2.0,” particularly in its emphasis on personalized health care and its participants (not observed in the Web 2.0’s democratized collaborations [13]). In addition, while we did not complete a systematic review of eHealth, previous publications have shown that the field emphasizes the “communicative foundations of eHealth and specif[ies] the use of networked digital technologies, primarily the Internet...for all stakeholder groups” [37]. As such, neither the stakeholders nor the principal tool used (the Internet) distinguishes Medicine 2.0 from eHealth. However, the principles of open source, generation of content by users, the power of networks, personalized health care, and the focus on collaboration across all stakeholders are not always highlighted by eHealth and suggest that these fields have different emphasis.

In addition, earlier in this paper we highlighted the issue that the technology based view of Medicine 2.0 (ie, the use of Web 2.0–like tools) could not clearly distinguish eHealth from Medicine 2.0. For example, we could conclude that every Internet health search using Google becomes a Medicine 2.0 search as the search algorithm is based on user-generated links. However, our definition implies that this cannot be taken for granted as Google does not meet many criteria of the Medicine 2.0 definition. First, it is not open; users do not have transparency on the algorithm or the ability to change it. Second, users do not have an intention to collaborate using Google or to help Google when assigning a link within a page. Rather, Google has commercialized a feature of Internet collaboration for its search and has not created a Medicine 2.0 collaborative platform. Despite this, other authors have argued that Google is the quintessential Web 2.0 company [38] and its use of network effects and user generated content will mean it will probably remain across the eHealth and Medicine 2.0 gray boundary.

Applying this definition to the original set of articles identified via key word searches on health and Web 2.0, we found that fewer papers were associated with the field. One main driver was the fact that the search terms (eg, “Medicine 2.0”) often found identified papers that had no relevance to the subject, though we did not bottom at the root cause of this effect. Others were relevant to eHealth in general, but not Medicine 2.0. For example, the study “Influences, usage, and outcomes of Internet health information searching: multivariate results from the Pew surveys” by Rice [39] provides detailed analysis on the use of the Internet in relation to health, but it does not address Medicine 2.0 issues specifically. A few papers ended on a similar gray boundary to that demonstrated by Google, such as Tse et al [40], and these were excluded from our review. This does not mean their findings are not relevant, but rather we found that the overall paper was not specific to Medicine 2.0 and should consequently be treated as an eHealth paper with potential implications for Medicine 2.0. 

Finally, we noted that rapid saturation in coding was achieved to obtain the salient themes used online. And while we believe this reflects a certain amount of common language used by the Health 2.0 or Medicine 2.0 online community, this does not mean all relevant themes were identified. For instance, social networking is only encapsulated in the “power of networks,” even though some authors would identify this as a very important separate trend and term. As such, this definition only identifies core or salient themes, not excluding other concepts, as being part of Medicine 2.0. We believe any compact definition will have difficulty in precisely delineating its complete scope.

### Research Question 2: Agreement

Is there agreement between online discussions and academic communities in their use of the term *Medicine 2.0*? If not, what does such divergence imply for future research? Regarding research question 2, we identified 56 articles in the research literature that covered four of the five major themes underpinning the Medicine 2.0 definition. In reviewing the literature and comparing it to prominent online themes, we found limited research into personalized health but did find extensive literature on methods and tools. We also noted potential overlaps with a separate body of research into open source health and a general call by authors for further research in specific areas.

Despite the fact that personalized or customized health is a key objective or benefit of Medicine 2.0 (based on online discussions such as those typified by [41]), no academic publications were found that focused extensively on this theme. Specifically, we believe researchers may need to look at how personalized online health care can evolve, such as the trade-offs between an active global site (with rich, regular but fairly uncustomized updates) versus a local site with very specific information to a regional context (but with less contributors and, hence, the risk of inaccuracy or less information).

By contrast, research responding to the tools and methods is the most extensive. In this theme, papers looked at the implications of a particular tool or method, such as the errors in user-generated content or the implications of open-source methods. For example, Deshpande and Jadad [42] offer an overview of the methods or drivers of Medicine 2.0, providing some support for our identified themes and definition. In addition to information inaccuracy and privacy, open-source methods have been widely studied within this theme relating to medical research. Examples can be categorized into two types: those that address the issues and benefits of a common license for the output of research (eg, [10,43]), and those that look at open-source methods to develop information technology tools for medical research (eg, [44,45]). However, open-source health is not subsumed by Medicine 2.0. The extensive literature on open source, such as the 3864 articles in PubMed as of February 2008, covers topics outside Medicine 2.0. For example, Hope [10] explores technology licensing not connected with Internet use, as opposed to Yang et al [46], who do consider a Medicine 2.0 open-source collaboration. As such, while Medicine 2.0 relies on open-source methods in health, and the topics overlap in areas, we believe care should be taken to view them as distinct research topics. 

Overall, there is a call for research in many areas, and Potts [47] suggests that researchers are significantly behind trends in eHealth and, more specifically, in Medicine 2.0 tools such as the use of blogs and wikis. Potts argues that extensive research is required to close this gap, which is supported by other researchers’ calls for more evidence to understand best practice models in using Medicine 2.0 for medical education and practice [48-51]. 

In addition to this call for research, we would expect publications on Medicine 2.0 to continue to grow in this theme for two further reasons. First, Web 2.0 tools are constantly evolving, and hence the impact of new tools will continue to require assessment. Second, two major tensions or research discussions exist that will also require investigation: information inaccuracy, and information privacy and ownership. We return to these tensions in the discussion on research question 3, and detail them in Table 5 along with how papers responded to salient themes. Note that some papers investigate Medicine 2.0’s impact for various stakeholders (indicated by “various” in Table 5), while others either touch on multiple themes or are difficult to classify (indicated by “over-arching or unclassified” in Table 5).

**Table 5:** Medicine 2.0 literature organized by themes and participants

Salient Theme	Year	Author	Principle Participant	Tensions
Over-arching or unclassified	2006	Skiba [52]	Researchers/scientists	Field’s existence
	2007	Manhattan Research [53]	Doctors	n/a
		Ferguson [54]	Patients/public health	Doctor’s concerns Privacy and ownership
	2008	Eysenbach [35,36]	Various	Field’s existence
		Versel [55]	Various	Field’s existence
		Guistini [56]	Various	Field’s existence
Tools and methods	2002	Burk [43]	Researchers/scientists	n/a
	2003	Killion et al [44]	Researchers/scientists	n/a
	2004	Boyle et al [45]	Researchers/scientists	n/a
	2005	Boulos et al [57]	Various	n/a
		Hope [10]	Researchers/scientists	n/a
	2006	Boulos et al [51]	Various	Information inaccuracy
		Boulos and Honda [58]	Various	n/a
		Castel et al [59]	Various	n/a
		Johnson et al [60]	Various	n/a
		Guistini [61]	Doctors	n/a
		Barsky [62]	Medical librarians	n/a
		Barsky [63]	Medical librarians	n/a
		Barsky and Purdon [64]	Medical librarians	n/a
		Karkalis and Koutsouris [49]	Patients/public health	Privacy and ownership
		Esquivel et al [65]	Patients/public health	Information inaccuracy
	2007	Boulos and Wheeler [47]	Various	n/a
		Liesegang [66]	Various	n/a
		Yang et al [45]	Researchers/scientists	n/a
		Saval et al [67]	Doctors	n/a
		Adams [68]	Patients/public health	n/a
		Boulos and Burden [69]	Patients/public health	Privacy and ownership
		Boulos et al [70]	Patients/public health	n/a
		Van den Brekel [71]	Patients/public health	n/a
		Barsky and Cho [30]	Medical librarians	n/a
		Barsky and Guistini [33]	Medical librarians	n/a
		Cho [72]	Medical librarians	n/a
		Connor [73]	Medical librarians	n/a
	2008	Eysenbach [74]	Patients/public health	Information inaccuracy
Collaboration and practice	2004	Eysenbach et al [75]	Patients/public health	n/a
	2006	Guistini [4]	Doctors	n/a
		Atreja et al [76]	Doctors	n/a
		Navarro et al [77]	Patients/public health	n/a
		Altmann [78]	Various	n/a
	2007	Bonniface et al [79]	Patients/public health	n/a
		Steyn and de Wee [80]	Medical librarians	n/a
		Mclean et al [50]	Doctors	n/a
		Potts [47]	Researchers/scientists	Field’s existence
Medical education	2006	Goh [81]	Patients/public health	Doctor’s concerns
		Boulos et al [82]	Various	n/a
	2007	Heller et al [83]	Patients/public health	n/a
		Crespo [84]	Patients/public health	n/a
		Skiba [85]	Nurses	n/a
		Skiba [86]	Nurses	n/a
		Skiba [87]	Nurses	n/a
		Skiba [88]	Nurses	n/a
		Sandars and Schroter [5]	Medical students	Doctor’s concerns
		Sandars and Haythornthwaite [89]	Medical students	n/a
	2008	McGee [90]	Medical Students	n/a
		Sandars [91]	Medical Students	Privacy and ownership

### Research Question 3: Tensions

What are the major tensions between the main stakeholders in Medicine 2.0 communities as identified by research? In relation to research question 3, four key areas of debate or tension between stakeholders were identified by our literature review:

1. The field’s existence: The definition of Medicine 2.0 and its existence as a legitimate research field, which this paper addresses, is an overarching issue, but it mostly concerns researchers.
2. Doctors’ concerns with patients’ use of Medicine 2.0, even if the information is accurate: This tension will mostly play out between doctors and patients in regular practice.
3. Information inaccuracy and potential risks associated with inaccurate Medicine 2.0 information: While this will concern all participants, it will be researchers, doctors, and patients who will have to understand the risks and techniques involved.
4. Privacy and ownership issues with Medicine 2.0–generated information: This may include such things as patient groups driving research agendas in addition to those sought by doctors and scientists.

The first main area of debate, an overarching theme, is related to the lack of agreement on what Web 2.0 is, and if it really exists [2]. Studies have generated justification for the study of Web 2.0 by the sheer size of its participants and the number of people who recognize it as a concept [92]. This debate has trickled into the Medicine 2.0 domain in discussions by people such as Skiba [52] and is continued by speculation that terms such as *Health 2.0* may be a fake “gold rush” [55]. The situation is complicated further by authors introducing Web 3.0 for medicine, speculating that some Web 2.0 tools such as social bookmarking will become redundant [56]. However, we believe people will continue to use Medicine 2.0 tools, and some researchers have argued that Medicine 2.0 and Health 2.0 may evolve into terms with relevance for different audiences, such as Medicine 2.0 as an academic and international focus, versus a business or consumer audience for Health 2.0 [35,36]. Our results neither confirm nor reject this hypothesis, but they do provide support for the idea that the terms currently have a high degree of overlap and that both are more complex than simply applying Web 2.0 to a health care context.

The second main debate surrounds collaboration and practise by doctors and patients. Separate to the issues of information inaccuracy, it encompasses resistance by some doctors to their patients’ use of Medicine 2.0. Their concerns arise from Medicine 2.0 causing unwanted behaviors in patients, such as not consulting a physician, consulting a physician too late, or coming to wrong conclusions about their disease management even if the information available to them online is accurate. The issue is not new and arose with eHealth. Ferguson [54] calls these doctors “e-Patient resistant clinicians” and suggests a sense of loss of control (and risk of being sued), paternalism, or lack of training driving these doctors’ behaviors. However, the issue is distinct in Medicine 2.0, where amplifying effects to this behavior are identified by certain authors, such as lack of training for doctors [5] or the difficulty of advising patients on use of Medicine 2.0 tools [81]. Overall, authors claim that doctors will need to recognize the emergence of Medicine 2.0 and that current training may not be sufficient to do so.

The third main discussion, based on the methods used to generate Medicine 2.0 information, is the risk of inaccurate online information. Misinformation has long been identified as a hazard of eHealth. However, studies have found little support for this concern [93]. These studies pre-date the rapid expansion in Medicine 2.0 use. Looking more closely at Medicine 2.0–specific information, Esquivel [65] notes the error and correction rate on an Internet-based cancer support group. The study found that most information was accurate and most false or misleading statements were rapidly corrected. Eysenbach [74] also examined the impact of information accuracy and credibility in relation to eHealth and noted that that patients will tend to use both intermediated (experts, authorities) and distributed (ie, Medicine 2.0) information to make their health decisions, thereby reducing any risk from inaccurate online information generated by users. In addition, apomediaries or gatekeepers acting at the network or group level work as collaborative filtering processes for distributed information that help users navigate through the onslaught of information afforded by networked digital media, reducing information risk further [35,36]. However, despite this early evidence of low risk, many practitioners and researchers remain to be convinced. This is demonstrated by responses to articles on Medicine 2.0’s potential, such as “the consequences could be disastrous for any inexperienced trainee following the advice” [4], or the need for authors to post a clarification after suggesting that Google could be used as a diagnostic tool [38].

The fourth and final debate is related to the consequences of the methods used to generate Medicine 2.0 information. Authors note that in addition to accuracy of information, privacy, ethical, legal, and ownership issues are also critical due to the nature of health information [49,69]. This applies not only to patients but to doctors who may use social networking sites for medical education and debate [91]. They suggest that potential models of identity management and authorization schemes should also be investigated in the context of Medicine 2.0 research. Once again, this tends to accentuate eHealth trends such as noted by Ferguson [54], who also highlights that those patient groups who run specific sites claim ownership over this data and are increasingly using it to influence the research agenda. Overall, new sources of health information are emerging via these methods, which will impact not only doctors who carry out research but could have potential implications for scientists working in the wider industry, such as pharmaceutical companies.

### Potential Limitations

Our study has several limitations that warrant attention. Clear risks arise from using Google and other search engines to define Medicine 2.0. First, in step 3 of the method, Google’s PageRank system may only identify popular self-referencing communities, which as noted by some researchers has bias against newer online content [16]. Second, search algorithms are rarely published, and hence we cannot be sure of the consistency of the counts, which has been subject to criticism at low levels for both Google and MSN searches [19,20].   

We mitigated the first risk via the iterative manner in which the definitions and themes were identified by comparing academic and online definitions for inconsistencies and by searching for theme rankings across all pages online to reduce the bias toward the popular pages. We did not find any major inconsistencies, even though the small differences in Medicine 2.0’s scope online and in academic publications were established. Examples include the online focus on personalized health and the lack of online focus on social networking, which has been identified as an important trend by other authors [35,36]. Other exact phrases that we anticipated but did not see included “semantic Web.” To mitigate their potential omission, we determined their ranking anyway, but due to lower rankings, they would not have emerged in the defining text of top salient themes used in our paper. This does not indicate that these are not very important themes, but rather that they are covered in more general concepts such as the power of networks.

We also examined the specific criticisms, such as Google returning inconsistent results below 1000 counts or Microsoft Live Search being inconsistent below 8000 counts and hence only ranked exact phrases above these levels. However, the use of different search engines further emphasizes that only the top exact phrases (eg, Blog or Podcast rather than Mashup) can be used with confidence to identify the salient themes as there was good agreement between search engines. Hence, we mitigated this risk by only using the top two to three and commonly ranked phrases, avoiding the bias that a term has been ranked highly only due to a particular search engine’s internal mechanisms.

### Concluding Remarks

Following the updated definition of Medicine 2.0, the literature describes five major themes: (1) the participants involved; (2) the impact on different collaborations and practice; (3) the ability to provide personalized health care; (4) the use in medical education; (5) its associated methods and tools.

There is now an emerging body of research into Medicine 2.0; in addition to the 56 papers we identified that address it directly, there are also many eHealth papers that have indirect implications for Medicine 2.0. Overall, they suggest that Medicine 2.0 will have a large impact on all areas of medical practice. Most of these publications are recent, since 2004, and call for more empirical research on various topics.

We expect research to continue to focus on the four major tensions between stakeholders that were found in the literature: the scope of the field including its definition and existence, the patient-doctor relationships impacted by Medicine 2.0, the methods and tools relating to information accuracy, and the methods and tools related to ownership and privacy. These issues are also found in eHealth; however, Medicine 2.0 is accentuating their impact. While touched on by some overarching publications, the lack of research into personalized health does not indicate that its importance is overstated by online discussions. Rather, we concur with other researchers who suggest that research currently lags behind practice in understanding the implications of Medicine 2.0.
